# Synthesis of student engagement with digital technologies: a systematic review of the literature

**DOI:** 10.1186/s41239-021-00270-1

**Published:** 2021-06-30

**Authors:** Larian M. Nkomo, Ben K. Daniel, Russell J. Butson

**Affiliations:** Higher Education Development Centre, University of Otago, Dunedin, 9016 New Zealand

**Keywords:** Student engagement, Digital technologies, Student learning

## Abstract

Restrictions on physical gathering due to COVID-19 has compelled higher education institutions to rapidly embrace digital technologies to support teaching and learning. While logistically, the use of digital technologies offers an obvious solution, attention must be given to these methods' pedagogical appropriateness, mainly how students engage and learn in the spaces supported by these technologies. In this context, we explored the degree to which digital technologies have contributed to teaching and learning practices over the past decade. The study employed a systematic review using a newly developed tripartite model for conducting and presenting literature review reports. The model approaches the literature review process systematically and employs three phases for the critical examination of literature: description, synthesis, and critique. The current review focused on student engagement across technologies that encompass social media, video, and collaborative learning technologies. Relevant articles were obtained from the Scopus and Web of Science databases. Three core themes were identified: there was no shared understanding of what constitutes student engagement with learning technologies, there was a lack of explanation concerning the contextual variation and modalities of student engagement across the digital technologies, and self-reporting was the primary method of measuring student engagement, rendering results as perceptual rather than behavioural. We argue that using multiple datasets and different methodological approaches can provide further insights into student engagement with digital technologies. This approach to measuring engagement can substantiate findings and most likely provide additional insights into students' engagement with digital technologies.

## Introduction

The contemporary higher education sector faces many challenges, including ability to meet the learning needs of diverse students and, student retention (Kahu & Nelson, 2018; Macfarlane & Tomlinson, 2017; Waldrop et al., 2019). These challenges are often linked to how institutions design their learning environments and engage students in their learning (Klem & Connell, 2004; Waldrop et al., 2019). Learning environments that support student engagement can influence the learning process (Kahu, 2013) and lead to the development of student critical thinking skills (Carini et al., 2006) and support retention (Waldrop et al., 2019; Wyatt, 2011).

Student engagement is a multifaceted and complex phenomenon to understand, however, it is considered a critical factor in supporting student learning and development (Kahu, 2013). With higher education rapidly deploying various forms of digital technologies into their learning environments, understanding how students engage with these technologies is critical to the design of flexible and highly adaptive learning environments that can cater to diverse student learning preferences. Also, understanding how students engage with digital technologies can enable educators to train students with various digital literacy skills and knowledge to support their learning.

Though the current generation of students entering university has a certain level of digital literacy, such literacy might be limited to engaging with entertainment technologies and games rather than using such skills  to acquire vital knowledge and skills (Prior et al., 2016). Since engagement is associated with academic achievement, researchers have identified various strategies to support better engagement (Barnacle & Dall’Alba, 2017; Kahu & Nelson, 2018; Koranteng et al., 2019). However, the meaning of student engagement means different things to different people (Kahu, 2013). Also, there is limited understanding of how students engage with learning technologies and the extent to which engagement with such technologies fosters enhanced learning outcomes.

This article surveys a wide range of studies published on student engagement with various forms of learning technologies  in the last decade (2010–2020). We conducted an in-depth analysis of the conception, meaning and nature of student engagement with digital technologies and how researchers measure, analyse, and present student engagement. The review focused on student engagement with three digital technologies (LMS, Social Media, and Lecture Capture). We believe this article will provide readers with an important reference point that provides insights into how students engage with digital technologies, and ways to design learning environments that are agile and cater to diverse student learning preferences. Four guiding questions were utilised to frame the research area for review as indicated below:

## The review guiding research questions:

1. What is the conception of student engagement with technology?
2. How are students engaging with various forms of digital technologies?
3. How is engagement with digital technologies measured?
  a Tools
  b Algorithms
  c Scales
  d Methods

4) What are the opportunities and challenges in measuring engagement in technology-enhanced learning environments?

## Methodology

This systematic review employed the systematic and the tripartite model. The model comprises a systematic approach to analysing and presenting the literature (Daniel & Harland, 2017). The model incorporates practical tools and strategies on how to write credible and critical reports. The model consists of three essential phases. The first phase of the model is deciding on articles to read, compiling summary abstracts and validating these with a mentor or peer. This stage is very similar to the procedure used in systematic literature reviews (Higgins & Green, 2008; Liberati et al., 2009).

In the first stage of the model, an investigation area is identified, and the researcher establishes the context and purpose of the review. The researcher further frames a research area for review and develops a search strategy, with explicit inclusion and exclusion criteria for selecting materials. This process should yield all the published material on a topic based on the criteria of interest. Once the purpose of the review is established, a search strategy is developed. The strategy involves the formulation of concrete search terms. It is essential to formulate relevant terms since this will determine the quality of resources identified (Boell & Cecez-Kecmanovic, 2015). The second part of the model is referred to as the 'tripartite approach'; it consists of three parts (description, synthesis & critique) and presents a model that combines the two stages as a structured and systematic guide. Tripartite I (description): In this stage, the systematic review presents a descriptive summary of the critical issues identified in the literature. This process provides the reader with an overview of developments in the field, the main areas of debate and the outstanding research questions. This is followed by the presentation of identified themes that have been carefully justified.

Tripartite II (synthesis): In the synthesis stage, the literature review goes beyond a description of what is published; it includes the synthesis and articulation of relationships between various published literature bodies. In this stage, the core focus is to synthesis ideas. This involves the extraction of the most important ideas or themes and a process of comparing and contrasting these to identify areas of similarity, difference and any controversies. This allows the researcher to clarify and resolve inconsistencies in thinking in the literature, thereby providing the best chance to make an original contribution to knowledge. Through synthesis, the researcher ensures that the particular problem of interest can be contextualised within the subject's historical context.

Tripartite III (critique): In the third part, the researcher reflects on the synthesis of the main ideas identified at the second stage to develop a critical view of the work reviewed in light of claims and evidence available. After a thorough description and summary, a critical thinking and judgment level can be applied in the review and presentation. Critical engagement requires the development of particular skills and strategies, and it mainly implies having the ability to examine claims against alternative evidence or views. It also requires a questioning mind and an openness to alternative views or evidence from other sources. The critique includes a positive dimension as the researcher aims to provide new ideas and alternatives.

## The systematic and the tripartite model

**Fig. 1:**
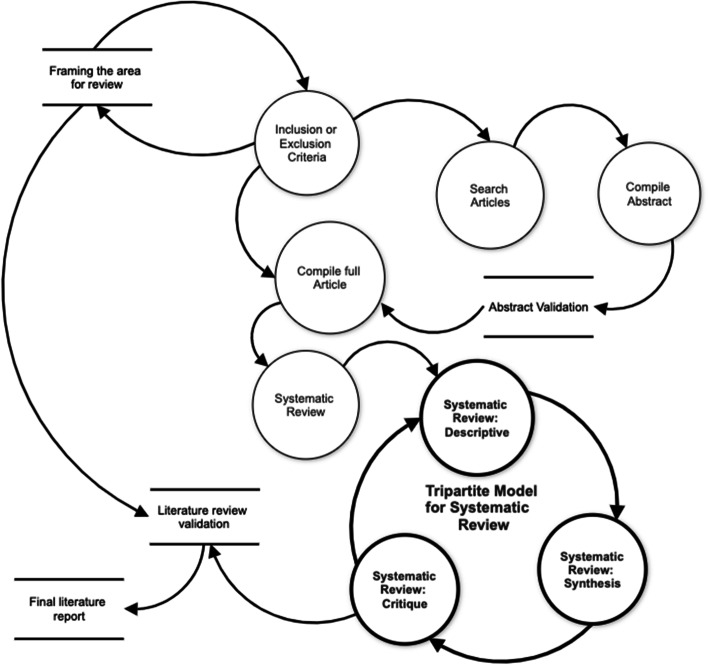
The systematic and tripartite model (Daniel & Harland, 2017)

When the two parts of the model are brought together, they describe an entire systematic approach and process to the literature review (Fig. 1). The model and step-by-step process components provide a checklist; however, the model also provides a schematic representation of the relationship between the different parts of the model.

## The initial search strategy

The initial stage was to establish the dominant digital technologies utilised in higher education to enhance students' learning experience and possibly their engagement. Therefore, the initial search aimed to answer the question: what technologies are students engaging with in higher education? To do so, a broad search string; (("student engagement" OR "learner engagement") AND technology AND ("higher education" OR "tertiary education" OR university)) was used. This search was conducted in the Scopus and Web of Science Databases. The string resulted in multiple records, as shown in Table 1, some of which were not relevant to the interest criteria.

**Table 1:** Initial search results

Database	Total results
Scopus	772
Web of science	463
Total	1235

The next phase involved conducting more targeted searches on student engagement with digital technologies. Similar to the initial search, Scopus and Web of Science databases were used to obtain resources for the review. The Scopus database was used as the largest abstract and citation database of peer-reviewed literature, scientific journals, books, and conference proceedings. The Web of Science database was also used since it provides an extensive set of world-class research literature from a rigorously selected set of academic journals that allows for the in-depth exploration of specialized sub-fields within an academic or scientific discipline (Li et al., 2018).

## Inclusion and exclusion criteria

Due to the rapidly changing nature of the field, the review included studies published between 2010 and February 2020. To ensure quality, only peer-reviewed papers were included in the review. The review's primary focus is to examine how engagement with digital technologies is conceptualised and the tools used for measuring it. In the context of this study, digital technologies is used to describe technologies utilised in student learning. Furthermore, the digital technologies examined were not specific to a particular field of study as long as they were within the context of higher education. Guided by the results of the initial search, the review focused on identifying how students engage with Learning Management Systems (LMS) (Blackboard), Social Media (Twitter) and Lecture Capture technologies (Echo360). Studies that were not relevant to the research questions were excluded. Overlapping studies were also discarded with the latest version of those being used. Studies with no authors were also excluded.

## Search strings

The final search strategy was refined to include three search strings (Search String 1–3 (SS1-SS3)). These were established to obtain the relevant articles to review. The strings were customized to meet the syntax of each database:

SS1: ("student engagement" OR "learner engagement") AND (LMS OR "Learning management systems") AND ("higher education" OR "tertiary education" OR university).

SS2: ("student engagement" OR "learner engagement") AND ("social media") AND ("higher education" OR "tertiary education" OR university).

SS3: ("student engagement" OR "learner engagement") AND ("lecture capture" OR "recorded lecture*") AND (higher education OR tertiary education OR university).

After running the search strings, the papers' abstracts were identified, read, and validated against the inclusion and exclusion criteria. The next step was to compile the full list of the articles, which were then systematically reviewed following the systematic and the tripartite model. In applying the systematic and tripartite model, the study utilised the within-study analysis, which involves the analysis of an entire article as well as the between study analysis, which consists of identifying the similarities and dissimilarities in the key findings from other literature (Daniel & Harland, 2017; Onwuegbuzie & Weinbaum, 2017).

The results of the search strings, preliminary selection and final selection are summarised in Table 2. The table shows the number of articles each string retrieved from the respective databases before validating them against the inclusion and exclusion criteria (N = 567). After validating against the search criteria and reading abstracts, a preliminary selection of (N = 189) was obtained. The next step was to then read through the articles for further validation and verification. This was done until the studies' findings became repetitive, which occurred after 30 articles had been reviewed. Therefore 30 articles made the final selection.

**Table 2:** Summary of the number of selected papers

Search string #	Total results from Scopus	Preliminary selection Results from Scopus after inclusion/exclusion and reading abstracts	Total results from Web of Science	Preliminary selection Results from Web of science after inclusion/exclusion after reading abstracts	Final selection
1	95	31	36	14	15
2	106	49	73	18	8
3	123	42	134	35	7
Total	324	122	243	67	30

## Findings

Findings from this review showed substantial research on student engagement. However, there is no consensus on what constitutes student engagement (Baron & Corbin, 2012; Harris, 2008; Kahu, 2013; Kahu & Nelson, 2018). The lack of consensus makes it difficult to ascertain the utility of engagement and its value in enhancing students’ learning experience and learning outcomes. The variation in the conceptions of student engagement has led to various discourses of different dimensions of student engagement (e.g. behavioural, social, and cognitive), though distinct from each other; these diemsnions of engagement are often used interchangeably (Burch et al. 2015; Christenson et al., 2012; Fredricks et al. (2016); leading to inconsistency in measuring  student engagement. Also, the lack of  a shared conceptualisation of engagement, makes it difficult to identify the semantic proximity between engagement and related concepts such as motivation. Alexander (2017) states, "when researchers do not explain their definitions of key constructs, they introduce a degree of conceptual ambiguity. And when the process of communicating theory or research starts with conceptual ambiguity, theory integration is far less likely to result." (p. 347). On the contrary, Christenson et al. (2012) view the lack of consensus as an opportunity to view engagement from different perspectives, enriching the concept's scholarly nature. A summary of the various conceptualisations of engagement is shown in Table 3.

**Table 3:** Summary: conceptualisation of engagement

Author(s)	Conceptualisation	Key constructs	Key assumptions	Examples
(Astin, 1984)	the amount of physical and psychological energy that the student devotes to the academic experience	Physical energy, psychological energy and academic experience	Engagement refers to the investment of physical and psychological energy Engagement occurs along a continuum The engagement has both quantitative and qualitative features The amount of student learning and development associated with an educational program is directly related to the quality and quantity of student engagement in that program The effectiveness of any educational practice is directly related to the ability of that practice to increase student engagement	Devotes considerable energy to studying, Spends much time on campus, Participates actively in student organizations,—frequently interacts with faculty members and other students
(Christenson et al., 2012)	Student engagement refers to the student's active participation in academic and co‐curricular or school‐related activities, and commitment to educational goals and learning	Active participation in learning, commitment to achieving educational goals	Multidimensional construct consists of behavioural, cognitive, and affective subtypes Active participation	Similar to the one above
(Harper & Quaye, 2008)	Participation in educationally effective practices, both inside and outside the classroom, which leads to a range of measurable outcomes	Participation in the education process,	In and out of class participation Measurable outcomes	Students actively participate in academic tasks regardless of being in a formal learning environment
(Kuh, 2016)	The time and energy undergraduates put forth in educationally purposeful activities combined with the policies, programs, and practices that institutions employ to induce students to put forth such effort	Time and energy are given to learning, Institution Induced	Time and energy Educationally purposeful activities Practices institutions employ to instigate students to exert effort	Students are responsible for their engagement, with the institution playing a part in providing the necessary environment for students to engage in
(Pekrun & Linnenbrink-Garcia, 2012)	A multi-component construct, the common denominator is that all the components (i.e., types of engagement) comprise active, energetic, and approach-oriented involvement with academic tasks	Active, energetic, thoughtful, involvement in learning	active cognizant approach to academic tasks	Students initiate their engagement and complete and participate in academic tasks due to this
(Coates, 2007)	A broad construct intended to encompass salient academic as well as certain non-academic aspects of the student experience	Academic and non-academic student experiences	Academic challenge Learning with peers Experience with faculty Campus environment	How the institution deploys its resources and other learning opportunities to get students to utilize their time and effort in these activities actively
(ACER, 2019)	Students' involvement with activities and conditions likely to generate high-quality learning	Student self-initiated involvement	Academic challenge Active learning Student and staff interactions Enriching educational experiences Supportive learning environment Work-integrated learning	Students actively interact with their learning environments in a manner that leads to learning
(Trowler, 2010)	Interaction between the time, effort and other relevant resources invested by both students and their institutions	Student and institution Investment in Time, effort and resources	Student and the institution interrelated components	How the institution deploys its resources and other learning opportunities to get students to utilize their time and effort in these activities actively
(Maguire et al., 2017)	Engagement is a product of the broader social and cultural context and not just the student's attribute	Students react to surroundings	Social Cultural context	Students react to the environment that is provided by the institution based on how the institution adapts its resources and teaching strategies to encourage participation
(Fredricks et al., 2004)	Malleable meta construct that is presumed to be based on the individual and the context	Individual and context-based	It can be changed Results from a variety of antecedents in the context, both social and academic, at both the school and classroom levels	Students engage based on what is availed to them
(Kahu, 2013)	Four views: Behavioural Psychological Sociocultural Holistic	Interrelates between student and institution	Students and University play a critical role	University and the student play a crucial role in student engagement with characteristics of both the university and the student as well as the relationship between the two is fundamental
(Filsecker & Kerres, 2014)	The sustained effort invested by an individual to manage and implement his or her intention of pursuing a previously chosen goal and entails cognitive, emotional, and behavioural components that reflect the individual's volitional/post decisional state	The sustained effort, self-imposed objectives	The student is crucial in their engagement	The students decide what to engage in
(Paulsen & McCormick, 2020)	Student learning relates to time and effort in studies by students; students benefit from environments that are collegiate and support their success institutions and faculty can facilitate effective educational practices in and out of the classroom	Time and effort from students, institutions set up effective educational practices to promote student success	The student invests time and effort into their studies in a conducive environment, set up by the institution	Students are responsible for investing in their engagement; the institution plays a part in providing the necessary environment for students to engage in
(Lawson, & Lawson, 2013)	Engagement is conceptualized as a dynamic system of social and psychological constructs as well as a synergistic process	Dynamic phenomenon, psychological and social, synergetic process	Requires social and psychology investment in energy	Collaborative, motivation to take part in the learning process

**Fig. 2:**
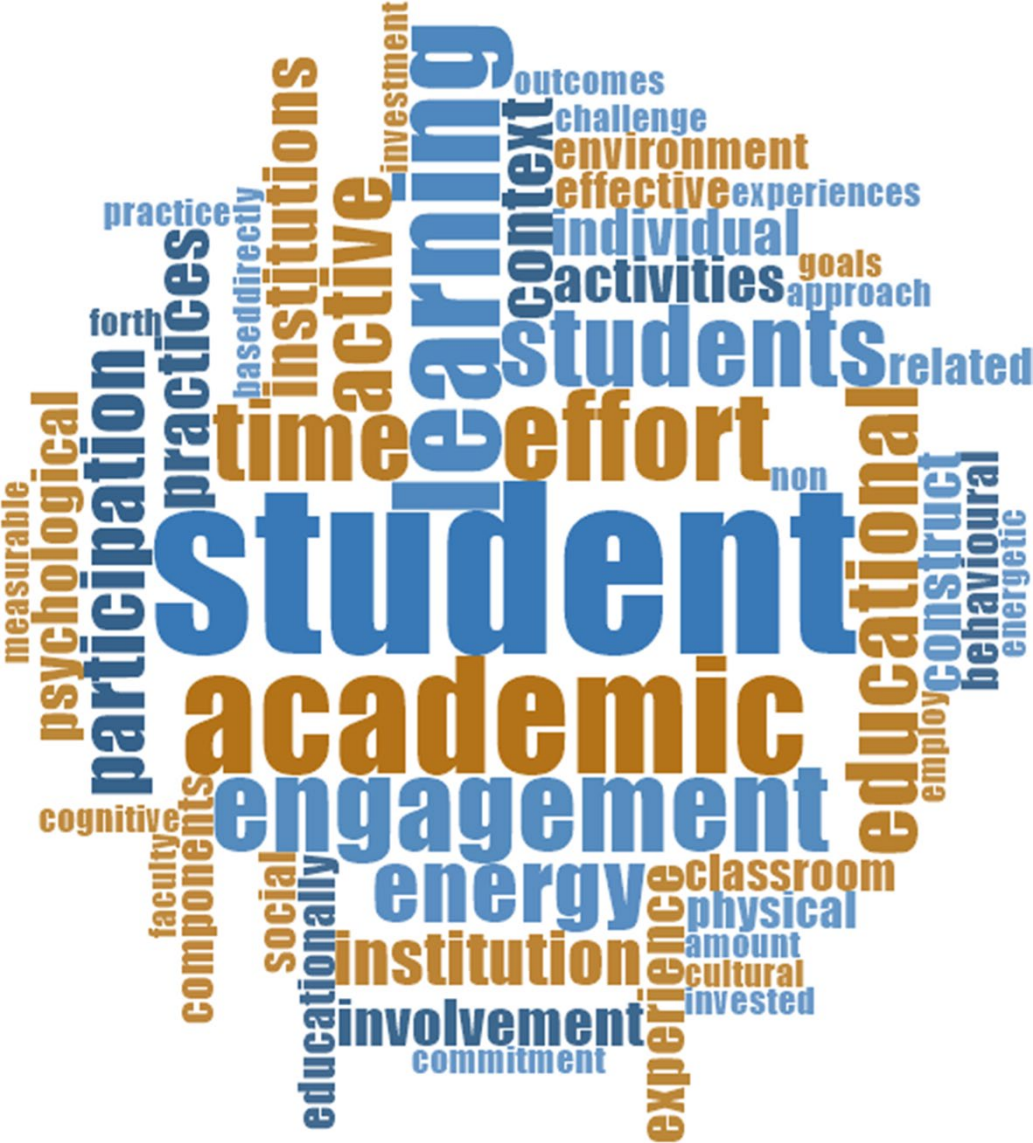
Frequent words in definitions

From the various definitions in Table 3, we extracted the fifty most frequently used  terms and  presented them visually sing a Word Cloud. The Word Cloud in Figure 2 shows various terms used to conceptualise the different dimensions of student engagement.

## The dimensions of student engagement

Student engagement is often conceptualised along three dimensions: behavioural, cognitive and emotional. The behavioural aspect of student engagement describes engagement along, effort and persistence in activities including extracurricular, social and academic. It is mostly concerned with getting involved in-class activities, completing given assignments, and regular attendance. Fredricks et al. (2004) stress that student engagement's behavioural aspect consists of positive conduct (non-disruptive behaviours or following stated rules).

Cognitive engagement refers to the psychological investment made towards learning activities, where the student is invested in learning activities. This dimension is exhibited when students perceive the value of what they are learning, understanding a topic and demonstrate a desire to learn and master skills. The cognitive type of engagement is linked to self-regulated learning, authentic intellectual capacity questions, focusing on tasks, and setting goals.

**Fig. 3:**
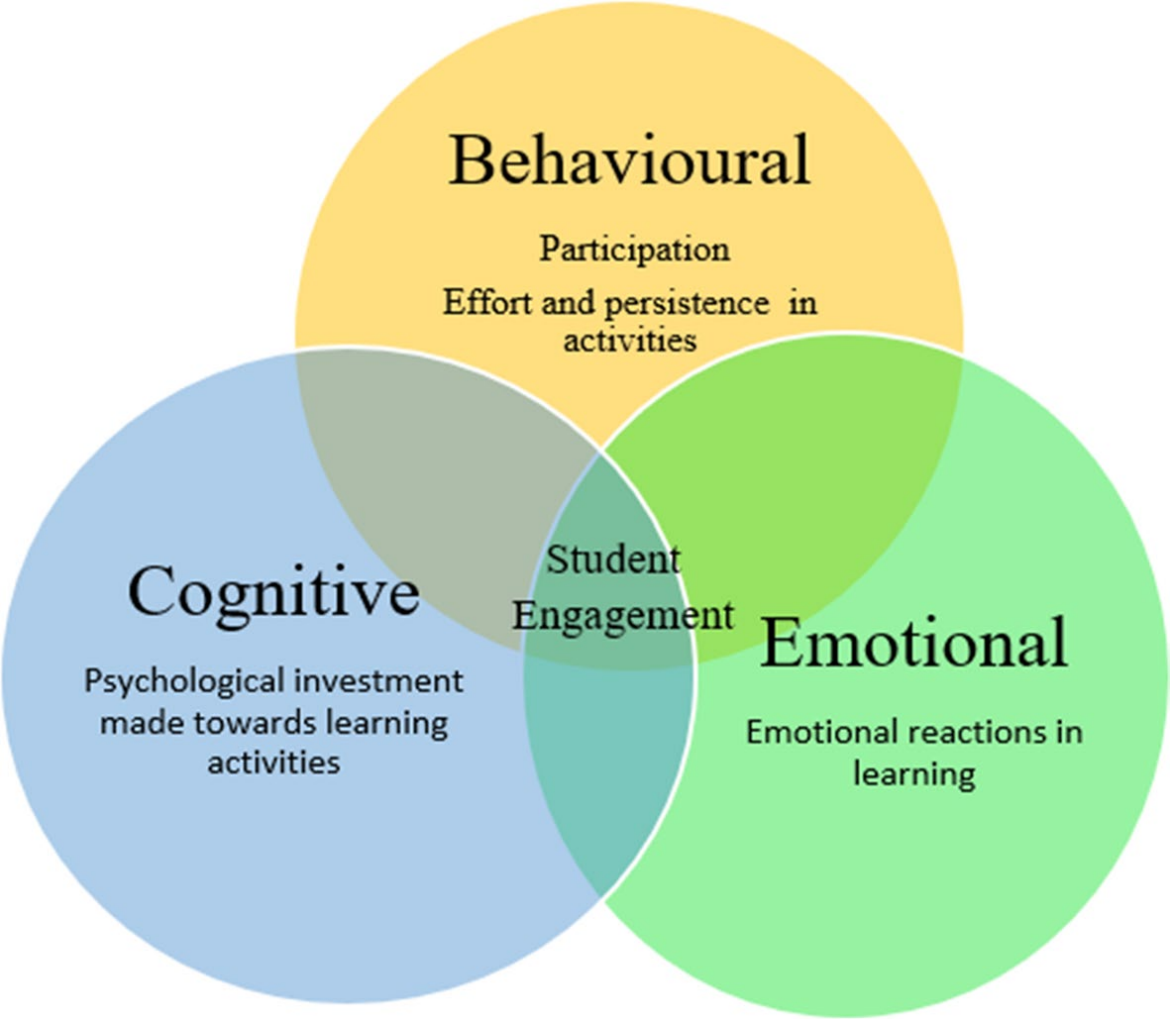
Dimensions of student engagement

Emotional engagement refers to emotional reactions (positive/negative) demonstrated in learning, such as showing interest, boredom, or anxiety towards their learning settings and feel like they belong in the school. The sense of belonging is considered vital to student's willingness to complete schoolwork (Baron & Corbin, 2012; Fredricks et al., 2016; Harris, 2008; Schmidt et al., 2018). The three dimensions are shown in Fig. 3 below. These dimensions are interrelated and contribute to a student's engagement.

Although each of the three aspects of engagement can be considered distinct, there is considerable overlap. For example, Filsecker and Kerres (2014) indicated that the behavioural part of the engagement that includes exerting effort and attention could be regarded as cognitive engagement. There are other engagement dimensions identified in the literature. Harris (2008) discussed academic engagement, specific to learning tasks, to move away from the general behavioural engagement that covers non-academic activities. Linnenbrink-Garcia et al. (2011) added social-behavioural engagement as a construct related to students affect and behaviour in collaborative group work (Fredricks et al., 2016). Reeve and Tseng (2011) propose the addition of agentic engagement to account for how students actively and constructively contribute to the learning environment. Agentic engagement factors in the student's ability to purposefully and proactively enhance the learning and teaching process. However, instead of a new dimension, it can be viewed as the union between the cognitive and behavioural dimensions.

Combining the three dimensions can provide a more in-depth description of students about their engagement (Fredricks et al., 2004). Therefore, it is important to measure all the dimensions when measuring student engagement because focusing on only one dimension can limit the understanding of student engagement. As behavioural, cognitive and emotional engagement interrelate in a volatile manner among individual students (Fredricks et al., 2004).

Critiques of behavioural engagement question whether participation in tasks can necessarily lead to desirable learning outcomes. For example, students in the class can focus on the instructor, which would be noted as engagement; however, the student's attention could be elsewhere (Linnenbrink & Pintrich, 2003). In other words, a student can be behaviourally engaged but not cognitively. Harris (2008) asserted that cognitive engagement seems to be the most linked to learning and that a student's physical participation does not necessarily assure cognitive participation. This is echoed by Linnenbrink and Pintrich (2003), who suggested that teachers need to engage students cognitively, not just behaviourally. This entails that instructors need to ensure that students deeply, critically and creatively think about the content being learned and reflect on what they know and do not know and utilise different learning strategies to help their understanding of the content.

The emotional engagement has also been contested as to whether students "feel good" about school learn (Skinner & Belmont, 1993). For example, students being enthusiastic in class does not necessarily translate to better learning outcomes.

Further, although research has claimed cognitive engagement to be the most important type of engagement, emotional and behavioural dimensions are seen as dimensions that may be required to enable cognitive engagement (Harris, 2008). For example, students need to be involved in the learning activity and, based on how they feel, then decide to engage cognitively. This goes further in underlining the importance and relationship between these three dimensions of engagement.

## Student engagement with learning management systems

Learning Management System (LMS) presents tools for collaboration, interaction, online course delivery, and reporting and tracking student activities (Rhode et al., 2017). LMSs are widely used in higher education institutions to support teaching and learning (Beer et al., 2010; Cabero-Almenara et al., 2019). University teachers employ LMSs such as Blackboard, Desire2learn, Moodle, Learning Space and next Ed to deliver course content and facilitate learning to students (Williams & Whiting, 2016; Zheng et al., 2018), and they provide instant and flexible access to content and teachers (Sánchez & Hueros, 2010). LMSs generate high volumes of data presenting numerous opportunities to extract useful analytics to support student learning. Analytics can also improve the teaching and learning process, enhance communication between the system users, and influence student outcomes (Bervell & Umar, 2017; Williams & Whiting, 2016). LMSs help manages large student groups and supporting advents such as distance learning (Zheng et al., 2018). Due to the dynamic nature of LMSs, it is essential to understand the extent to which they support student engagement to lead to better learning outcomes. LMSs can incorporate various forms of synchronous and asynchronous tools. Tools such as discussion forums and chats can be used in real-time for synchronous activities and may also be used asynchronously.

## Student engagement with LMS: descriptive

Studies on LMS student engagement have identified the influence of behaviours on achievement (see Avcı & Ergün, 2019; Umer et al., 2018). However, LMSs are dynamic environments where students can exhibit various forms of engagement. For example, emotional engagement may be fostered through chat platforms and discussion forums within LMSs. This can help students to connect and create a sense of community. Students may also engage cognitively in LMS through problem-based learning activities and self-regulated learning through the access of resources at their own pace.

However, effective students' engagement with LMS is dependent on how students and instructors utilise LMS. Klobas and McGill (2010) investigated the role of student and instructor involvement in LMS success. They found that when instructors provide regular guidance to students in LMSs, students are likley to gain improved effectiveness and productivity when studying. 

Little-Wiles and Naimi (2011) looked at what educators can do to ensure students are fully engaged when interacting in LMS. They found that students use LMSs to create self-awareness of learning, e.g., checking one's progress and requirements of a course and communicating with their peers.

A behaviourism perspective tends to explain better the various forms of student engagement in LMSs. For instance, this can be seen from students' navigational pathways in LMSs (observable change in behaviour), depending on how the LMS is set up by instructors (the stimuli). Corrigan et al. (2015) explored the impact of presenting students with their engagement data in a VLE to determine trends linked to student attainment. The study found students who received notifications on their engagement with the VLE, compared to non-participants in the various courses, showed improvement in their grades.

Though studies look at engagement in LMS from the behavioural perspective, Henrie et al. (2018) scrutinized the relationship between student activity log data in LMS and self-reported student engagement survey scores, intending to understand whether or not LMS log data could be used as a proxy measure for students emotional and cognitive engagement. The study did not find any significant relationship between the use of log data and students self-reported emotional and cognitive engagement. This underscores the relationship between observed and reported states of engagement.

LMSs provide tools to track engagement through tools such as the Moodle Engagement Analytics Plugin (MEAP) and Blackboard Analytics. The MEAP monitors student behaviour on three tasks: forum activity (if students are participating in the forum), login activity (the duration frequency and time of login) and assessment activity (if submissions are made on time) (Liu et al., 2015; Luna et al., 2017; Yassine et al., 2016). However, the drawback of MEAP is that lecturers need to enter thresholds for each of the three entities, which some lectures might see as an issue. Similarly, Blackboard Analytics monitors students engagement patterns, evaluates learning outcomes, and assess the use and adoption of online learning tools (Jones, 2012). Unlike MEAP, Blackboard analytics provides a more holistic approach in analysing students data by including data such as demographic data and previous course data (Whitmer, 2015). However, this requires integration with the institutions' student information system, which may have some privacy concerns.

## Student engagement with LMS: synthesis

LMS is often used in conjunction with face-to-face lectures to enhance student learning (Barua et al., 2018; Graham et al., 2013; Venugopal-Wairagade, 2016). However, it is also used in distance education programmes (Altunoglu, 2017). Effective use of LMS leads to enhanced learning. However, analysis of the literature suggests that the instructor's role in facilitating engagement and learning within an LMS is critical to students success (Baragash & Al-Samarraie, 2018; Barua et al., 2018; Klobas & McGill, 2010; Little-Wiles & Naimi, 2011). In order for LMS implementation to be successful, both students and instructors need to play their part in the process. For instance, the instructor’s involvement in LMS course design can benefit students by integrating interactive course designs that allow collaboration and communication (Swart, 2015; Wang, 2017).

Findings from this review suggest that engagement in LMS is predominantly behavioural oriented, where students are expected to respond to a stimulus (the LMS environment) set up by an instructor (Barua et al., 2018; Little-Wiles & Naimi, 2011; Venugopal-Wairagade, 2016). Most of the LMS actions, such as logging on, posting on forums, accessing learning resources, and assignments, are behavioural traits and would mostly favour the behavioural dimension. Several studies have revealed that students' engagement in LMS can be influenced by demographic characteristics such as age, digital literacy and educational background (Baragash & Al-Samarraie, 2018; Klobas & McGill, 2010; Swart, 2017; Venugopal-Wairagade, 2016). For example, although most students are technologically astute, their digital literacy may be limited to digital learning environments (Prior et al., 2016). Therefore, students' different experiences can lead them to engage differently with LMS.

Most studies have approached the measurement of engagement with LMS through students self-reporting measures, such as questionnaires (Barua et al., 2018; Klobas & McGill, 2010; Little-Wiles et al., 2010; Venugopal & Jain, 2015). However, most of these questionnaires span across disciplines and are generally produced as a one-size-fits-all instrument. They are unlikely to capture engagement as teaching and learning genuinely are likely to differ across disciplines.

LMS generate data based on user actions. The use of analytics for analysing these data may be more insightful (see Liu et al., 2015; Luna et al., 2017; Messias et al., 2015; Yassine et al., 2016). Some studies have utilised questionnaires and system logs to measure engagement (see Baragash & Al-Samarraie, 2018; Henrie et al., 2018; Wang, 2017). These studies found a positive relationship between engagement with LMS and achievement (see Baragash & Al-Samarraie, 2018; Wang, 2017). However, Henrie et al. (2018), focusing on emotional and cognitive engagement, found logs inefficient as a proxy for engagement. Studies utilising logs independently have also found them indicative of how engagement affects achievement (see Swart, 2015; Umer et al., 2018). Research suggests discussion forums, frequency of logins as well as submission activities are the most common data for analysing engagement in LMS (Henrie et al., 2018; Liu et al., 2015; Luna et al., 2017; Messias et al., 2015; Swart, 2015; Venugopal & Jain, 2015; Yassine et al., 2016). Wang (2017) indicates it is relatively easier to measure and collect behaviour engagement in LMS. Moreover, when students initially engage with LMS, it is highly dependent on how the instructor sets it up; therefore, measuring behaviours alone is most likely the best way to understand engagement with LMS.

## Student engagement with LMS: critique

Based on how different research studies have approached student engagement, it is apparent that the construct is complex and multifaceted. With no consensus on what constitutes student engagement, some studies may have misrepresented measurements of engagement dimensions. The factors used in some studies do not necessarily well represent the dimensions of engagement they claim to measure. Furthermore, studies can define and measure the same dimensions differently, thus creating an overlap in clarity. In some cases, the methods used to measure student engagement may not genuinely reflect student engagement's accurate measurement. For example, observation studies where the students know they were being observed can suffer from the Hawthorne effect as students change their behaviour due to knowing they are being followed. The use of survey instruments alone also limits our understanding of student engagement as they are limited to collecting perception data.

Furthermore, the use of analytics without context can further limit our understanding of student engagement. For example, measuring clickstream data can be inaccurate as a click does not necessarily equate to engagement. A click to download a document may not mean the same engagement as posting a discussion on the forum. Factors such as prior knowledge, technical ability, and student's motivation to learn, among others, can influence the level of student engagement within LMS as well as student's intentions to engage with an LMS; however, as LMS engagement is mostly measured in behaviours, most studies do not include these factors. Moreover, though there is a general belief that most students are digitally literate, it is crucial to assess the level of digital literacy of students as students may not engage with LMS due to digital illiteracy. The failure, in some instances, to account for students across various disciplines when measuring engagement can further limit our understanding. For example, students from some fields can be more inclined to engage in specific ways with LMS technologies than other students based on their domains of study. This can be due to students having different task-based interactions with LMS making their engagement vary. In terms of datasets, most studies utilise datasets that are convenient as small samples ranging from single courses with a few students and one instructor to multiple courses are typically used (see Corrigan et al., 2015; Klobas & McGill, 2010; Little-Wiles & Naimi, 2011; Swart, 2017; Umer et al., 2018). Therefore, it can be difficult to infer causation from cross-sectional data. The results of some studies are therefore applicable only to specific cohorts.

Further, studies that do not utilise control and treatment groups in studies with comparisons can have somewhat less reliable results. In general, most studies in LMS measure the behaviours of students to infer student engagement. This, however, leaves questions such as if these measurements are accurate reflections as they do not include the other dimensions of student engagement.

## Engagement with social media

Social media use in education has increased rapidly over the years (Esteve Del Valle et al., 2017). Both students and instructors have taken advantage of social media in education (Junco et al., 2011). Social media facilitates social learning, improved self-confidence and communication between students and instructors, which are benefits associated with the active use of education (Nkomo & Nat, 2017). Junco (2012) examined the relationship between Facebook use and student engagement, defined as the time spent preparing for class (academic engagement) and time spent in co-curricular activities (co-curricular engagement). Findings suggest that students' involvement in Facebook can either positively or negatively engage with their education.

Similarly, Williams and Whiting (2016) explored students' use of Twitter in enhancing engagement. The study noted, "some define engagement as the frequency with which students participate in activities that represent effective educational practices and conceive of it as a pattern of involvement in a variety of activities and interactions both in and out of the classroom and throughout a student's college career. Additionally, the phrase "student engagement" has come to refer to how involved or interested students appear to be in learning and how connected they are to their classes, institutions, and each other" (Williams & Whiting, 2016, p. 312). The study indicated students felt more engaged when twitter and the LMS were used. The use of Twitter also had a positive relationship with students' perceptions of engagement in the marketing course. Seniors students were found to use the LMS more frequently than their junior counterparts, and no difference in the use of Twitter. Furthermore, there was no difference between junior and senior student's engagement levels.

Alshuaibi et al. (2018) stated that social media could enhance student's cognitive engagement in learning as they found cognitive dimension had a mediating role in the relationship between social media and academic performance. Fagioli et al. (2015) analysed the use of a social media site and learning outcomes regarding community colleges. The study found a relationship between social media use and academic outcomes. Students who are actively engaged in social media tend to perform better in their learning outcomes than inactive students. Furthermore, the posted comments and discussion's quality and relevance was a significant factor in sustaining the application's continued use. Suggesting students find value in meaningful peer discussions.

Saunders and Gale (2012) noted that students are less engaged in large lecture halls and hardly ask questions. Ellis (2015) suggested that the use of Padlet as a social media tool that allows students to post comments on an online wall can enhance the learning experience students as they engage with materials posted by other students Tiernan (2014) found Twitter enabled students to contribute to discussions in a less intimidating manner and enabled engagement with peers and course content (Soluk and Buddle (2015). Ally (2012) further indicated the ability of social media to promote engagement through collaboration and communication, similar to Ellis (2015); Soluk and Buddle (2015); Tiernan (2014). Ally (2012) found most participants embraced Twitter as an enhancer to collaboration and communication in the classroom. The study further noted increased class participation levels, attentiveness, and engagement compared to previous years, where traditional means were used to encourage interaction. This suggests students find the use of social media for interaction to be fulfilling for them.

## Engagement with social media: synthesis

Students use social media as a way to improve their interaction between lecturers and peers. More specifically, social media such as Twitter and Facebook positively impact students' engagement with peers and instructors (Ally, 2012; Junco, 2012; Tiernan, 2014; Williams & Whiting, 2016). Several studies looked at how students utilise social media to enhance their learning experience and engagement (Ally, 2012; Alshuaibi et al., 2018; Ellis, 2015; Fagioli et al., 2015; Junco, 2012; Tiernan, 2014; Williams & Whiting, 2016). Studies have also related engagement with social media and positive influence on academic outcomes (Alshuaibi et al., 2018; Fagioli et al., 2015; Junco, 2012). When discussions inherent in social media are of good quality and relevant, this leads to sustained interests in social media use, which can positively influence student learning outcomes (Fagioli et al., 2015). Lack of confidence and boredom are issues students face in traditional lectures. Social media has been used to try and avert this by allowing students to use social media to post questions for discussions in a non-intimidating manner with students (Ellis, 2015; Tiernan, 2014).

Like LMS, most of the studies used quantitative approaches relating to usage, such as the number of posts, frequency of posts, etc., mostly associated with behaviour (Fagioli et al., 2015; Junco, 2012; Tiernan, 2014; Williams & Whiting, 2016). The use of self-reported measures such as questionnaires to examine if social media enhances student engagement and learning experiences is prevalent (see Junco, 2012; Tiernan, 2014; Williams & Whiting, 2016). Further, statistical methods such as t-test, regression and descriptive statistics were used for analysis (Alshuaibi et al., 2018; Ellis, 2015; Junco, 2012). Some educators have made social media voluntary in their classes (Tiernan, 2014; Williams & Whiting, 2016). Some have made activities that are graded to be conducted on social media (Soluk & Buddle, 2015). Similar to studies measuring engagement in LMS, behaviours from self-reported measures and quantitative analysis are utilised. However, as a tool that supports interaction, social media can facilitate other dimensions of engagement, such as emotional and cognitive engagement. Therefore, other measures such as content, thematic and social network analysis can provide more insights into student engagement with social media.

## Engagement with social media: critique

The use of small samples that utilise a single course or a few students to analyse student engagement with social media can make some of the results challenging to take at face value, as they are less generalizable (see Ally, 2012; Ellis, 2015; Soluk & Buddle, 2015; Tiernan, 2014). Studies that use self-reported measures in isolation are limited regarding the data they can obtain as it is perception data. Moreover, the data can be biased, for example. The use of questionnaires in classrooms can be disruptive as students then have to stop academic tasks to concentrate on these. Furthermore, behavioural indicators are the most collected; however, social media can promote other student engagement dimensions, which cannot necessarily be inferred through behaviours. For example, Twitter has character limits, requiring users to write concise, well-thought posts. Furthermore, students tend to want to represent themselves as best as possible on social media and therefore put some thought into what they write.

The conception and discourse with social media also seem to be over generalised as social media refers to one form of technology. There is also no transparent pedagogical approach to using social media to enhance student learning as the uptake and utilisation vary effectively. Therefore, the efficacy of social media as a platform for learning is not apparent. Furthermore, there is no clear indication of social media providing anything further than a forum for students to discuss. Therefore, clarity on how social media can facilitate different types of student engagement is scarce. Away to understand engagement with social media in depth and analysing users' behaviours would be to examine the content that students post. This would possibly help identify the emotional and cognitive dimensions. Further analysis could be based on social network analysis.

## Engagement with lecture capture

Lecture capture technologies are designed to capture individual or all elements of a live lecture in digital format. It can either be audio in the form of a podcast or a video with audio; other systems are capable of recording the images on a computer or document camera and audience audio and video (Newton et al., 2014). Studies have found live lecture attendance vital as students who attend their lectures tend to perform well (Greener, 2020; Zhoc et al., 2019). Therefore, the use of lecture capture is contentious because some educators fear that once students are provided with lecture materials, they can refrain from attending live lectures. However, it can be argued that even if students reduce their attendance in live lectures, viewing lecture recordings can be a proxy to attendance.

McGowan and Hanna (2015) examined the impact of recorded lectures on attendance and student attitudes. The study investigated students' behavioural viewing patterns, which can be interpreted as the behavioural engagement dimension and the rewind and replay actions of lecture recording, which can be regarded as a form of cognitive engagement. The findings suggested the videos did not affect student's attendance; students also stated that the videos could not replace their face-to-face lectures and were beneficial to learning. Moreover, viewing patterns were higher in the early stages of the course, with shorter videos and assessment-related videos as students indicated they used they primarily accessed videos for revision purposes. This was further demonstrated by students volatile rewinding and replaying, which could have been confusion, interest, or engagement.

The use of lecture recordings allows students to engage with lecture content at their own time and pace and engage with resources in ways that suit them. These findings are further outlined by Chapin (2018); Dona et al. (2017); Draper et al. (2018).

Chapin (2018) surveyed Australian undergraduate psychology students who used lecture recordings to prepare for exams, prepare for study notes during a semester, catch up on missed lectures, and obtain clarity on the lecture's ambiguous parts. Students' final grades were the same regardless of low or high access to recordings or low or high attendance in lectures. This indicates the flexibility of WBLT, and how students may engage with it in different ways, with similar academic achievement.

Draper et al. (2018) examined the extent to which Law instructors use lecture recordings and how undergraduate students perceive and engage with lecture recordings. Findings indicated some staff suggested that lecture recordings could benefit the students and that the recordings did not affect students' attendance. Most students also demonstrated their attendance was not affected. The study indicated that students used the recordings as a supplementary tool to organise their notes, for catching up when a class was missed, and preparing for exams. An improvement in positive study activity overtime was also noted.

Dona et al. (2017) investigated how undergraduate students experienced a fully integrated lecture recording system across several disciplines. The study concluded that students were generally positive about the value of lecture recordings. The findings were substantiated by Chapin (2018), who indicated that students use lecture recordings to clarify confusing topics, prepare for exams, learn at their own pace, improve their learning experience, and help balance their schedules between their studies and other obligations. Most lecturers were undecided about the value of recorded lectures. Therefore, differences in lecturer-based engagement were noted based on disciplines. Lecturers in business and social science were more positive towards the recorded lecture system than lecturers in the engineering and science disciplines. The findings from Dona et al. (2017) raise the question on the idea of a "one-size-fits- all" lecture recording system as differences in discipline lecturer styles and approaches to teaching are noted. They were indicating that not all students may engage with lecture recordings the same.

Further Trenholm et al. (2019) investigated undergraduate mathematics students' cognitive engagement with recorded lectures. The study approached cognitive engagement via two scales on measuring learning approaches: surface and deep. The study found that the combination of a decline in lecture attendance and reliance on recorded lecture videos had an association with an increase in surface approaches to learning. Edwards and Clinton (2019) examined the usage and impact made by introducing lecture capture in a Bachelor of Science programme course. The study found; the impact of lecture recordings was negative as students live lecture attendance dropped. They illustrated the drawbacks of over-reliance on lecture capture as a replacement for attending lectures as attendance is seen as an engagement indicator. However, viewing the lecture recordings had no significant association with attainment. Moreover, the study indicates lecture capture availability will most likely negatively affect less engaged students who might utilise more of a surface learning approach.

Studies suggest a need to address best practices in using recorded lecture videos not only in mathematics but possibly in other fields as well Edwards and Clinton (2019); Trenholm et al. (2019). The Instructional design of lecture recordings can influence best practices for utilising lecture recordings. Costley et al. (2017) examined the relationship between instructional design and student engagement in video lectures. The study outlines five instructional design indicators that can lead to the watching and completion of videos. These are utilizing the medium, establishing netiquette, establishing time parameters, setting the curriculum and design methods. These five elements aim to provide students with a clear pathway to success in the online learning environment. Instructional design is present when students view these elements as enhancing their engagement and learning. Findings suggested that the videos' design does influence students' engagement, and therefore instructors should pay attention with regards to how their courses are designed. Moreover, Seifert (2019) aimed to identify students' learning preferences as well as their attitudes with regards to using recorded lectures and how this affected student attendance in lectures. The findings indicated students had a positive experience that aided them in understanding the learning materials, as the lecture recordings met students' various needs.

Ebbert and Dutke (2020) identified five clusters of students based on how they utilised lecture recordings. The study outlines behaviour variables representing lecture capture, usage frequency, selective and repetitive watching, live lecture attendance, social context and location. These variables can represent the behavioural and cognitive dimensions of engagement. The clusters in descending order of size were frequent repetition, Selective repetition, Frequent consultation, Selective consultation and Increased absenteeism. These clusters indicate students engage differently with lecture recordings; therefore, strategies should be generated to support the different ways students engage with lecture recordings. Lecture recordings are a flexible platform concerning how students engage with them. Therefore, they keep a flexible type of engagement that enables students to utilise lecture recordings according to their preferences. Although most studies address the behavioural dimension, lecture recordings can facilitate other dimensions of engagement. The common use of self-reported measures alone also limits how students engage with lecture recordings as these obtain student perceptions.

## Engagement with lecture capture: synthesis

Many educators believe that recorded lectures are likely to influence lecture attendance (McGowan & Hanna, 2015). Studies that explore how students engage with recorded lectures reported mixed findings, with some indicating that attendance has dropped due to the use of recorded lectures (Edwards & Clinton, 2019), while others seem to suggest that the availability of lecture recordings to students does not affect lecture attendance (Seifert, 2019). However, lecture recordings offer students flexibility as they can engage with them in various ways, such as taking notes, catching up when a class was missed, and preparing for exams (Chapin, 2018; Dona et al., 2017; Draper et al., 2018). Furthermore, the studies have mostly looked at the usage of the recorded lectures from the perspectives of behaviours (Chapin, 2018; Dona et al., 2017; Edwards & Clinton, 2019).

Further several of these studies utilized self-reported measures in the form of questionnaires and interviews (Chapin, 2018; Costley et al., 2017; Dona et al., 2017; Draper et al., 2018; Ebbert & Dutke, 2020; Palmer et al., 2019; Seifert, 2019). However, studies such as Edwards and Clinton (2019) McGowan and Hanna (2015) have used trace data combined with other data sets to a more significant effect. The data sets used have mostly been small and covering single cohorts (Draper et al., 2018; McGowan & Hanna, 2015), although some have used more diverse cohorts (Dona et al., 2017). Common methods used to analyse the data have been statistical methods such as Chi-square, descriptive statistics, t-test, and regression (Palmer et al., 2019; Seifert, 2019; Trenholm et al., 2019). Most of the focus on lecture recordings has been on their effect on attendance in live lectures; however, they provide flexible engagement opportunities to students. More emphasis should move to their efficacy as a learning resource that enhances student learning. Furthermore, other features such as supplementary note taking features should be analysed for insights.

## Engagement with lecture capture: critique

Students generally advocate for lecture recordings to be made available as they value them highly. However, there is not much work done on moving forward and providing best practice strategies for student engagement with lecture recordings. Therefore, the pedagogical efficacy of lecture recordings is not overtly apparent. Furthermore, most studies have mostly looked at the effect of student engagement on one proxy: live lecture attendance. Therefore, not much has been addressed about most learning outcomes.

Furthermore, it is not clear how different student engagement types interrelate and can be facilitated through lecture recordings. Small and homogeneous samples are mostly used, which may fail to account for diversity among students. The design of the recorded lectures may be one reason for the mixed results as the design has been found to affect engagement, with shorter videos seen as more engaging (Costley et al., 2017). However, most studies do not consider design. Studies that utilised different approaches such as analytics only analyse behavioural data such as the total number of views. Although lecture capture views go through peaks and declines, there is no general understanding of why this is the case.

Furthermore, the methods used to infer videos as being watched somewhat rely on assumptions as it is not guaranteed that students watch the recordings they play. The use of courses from single instructors provides results that leave pending questions challenging to generalize. The emphasis on measuring the impact on mostly students’ live lecture attendance has left a limited understanding of student's engagement with lecture recordings. Furthermore, more insights can be obtained by utilising different datasets, including those obtained from trace data sets. This can help identify other dimensions of student engagement to understand student engagement with lecture recordings better.

## Discussion and conclusion

The articles reviewed in this paper have showed various research on students’ engagement with digital technologies. The review revealed a lack of shared understanding of what constitutes student engagement in learning, let alone student engagement with digital technologies. The lack of shared understanding has led  to the use of different techniques and measures to understand student engagement. The variation in meaning and measures reinforced the prevalence of the diversity of perspectives in student engagement literature. With no clarity in meaning, studies have used different variables and dimensions to measure engagement. For example, participation has been used as a proxy variable for measuring student engagement with digital technology through clickstream data, which provides a limited view on engagement. Furthermore, the behavioral, social, and cognitive aspects of engagement remain the dominant dimensions of engagement in the literature.

Studies that have operationalised student engagement have mostly addressed one or at most two of the dimensions. While widely used, this approach fails to validate the interrelation of the three common dimensions of engagement (social, emotional and cognitive). Furthermore, measuring one dimension on learning outcomes does not provide a holistic view of student engagement. The behavioural dimension is the common dimension addressed in the literature, mostly due to its traceable action. This negates the emotional and cognitive dimensions. The emotional and cognitive dimensions remain challenging to directly observe, hence self-reported measures are increasingly used. However, some studies have utilised behavioural trace data as proxies to a certain degree of success. Studies have indicated psychological engagement can lead to behavioural engagement. Further exploration of how the three different dimensions of engagement can be measured together is crucial to understanding engagement. It is also important to examine the extent to which psychological attributes of engagement influence the behavioural dimension of engagement.

Several studies utilise convenient sampling technique when examining engagement, as the samples used were mostly from single courses and in a particular discipline, making the generalisability if the results limited. Further, the use of cross-sectional design in some of the studies examined are limited in their ability to explaining factors that can contribute to our understanding of engagement broadly and how it can be supported in digital learning environments. We argue that a more holistic approach that would incorporate participants from more diverse domains may yield a better understanding of how students with different demographic characteristics studying different subjects engage with digital technologies.

 The cohort of students entering higher education are digitally savvy with different levels of technology literacy, therefore it is essential to incorporate demographics for richer insights in understanding how students engage with learning technologies. 

In the studies reviewed, most approaches to the measurement of student engagement rely on self-reported measures, this is a concern, as some students may not recall their pre-self-report actions. Further, the use of a single source in the form of questionnaires mostly used in the literature is liable for single-source bias. Some studies have used learning analytics approaches that are less intrusive than self-reported measures. However, these have been mostly conducted in a short timeframe making durable patterns difficult to establish.

Studies can look to use larger samples, and accounting for more variables as noted by Helal et al. (2018), institutions may look to undertake the complex task of understanding student academic performance predictors, which may be affected by numerous factors such as the economic, social, demographic, cultural and academic background. Student engagement is similar in its complexity of variables that may affect it. One may even argue that addressing those multiple factors at the engagement level can help understand student outcomes. In particular, the use of perception data alone for student's engagement with digital technologies limits our understanding of student engagement in these environments. With most studies skewed towards a perceived behavioural dimension of engagement, it can be fruitful for researchers to incorporate different data sets to the more traditional data sets, such as trace data, as most digital technologies generate data in their system logs. In conclusion, the following outstanding issues need to be addressed:

## Outstanding issues in the studies examined

- There is no simplified shared understanding on the meaning of student engagement, let alone student engagement with learning technologies.
- Current research on student engagement does not adequately describe the contextual variation and modalities of student engagement with various forms of digital technologies.
- The conception and discourse with particular forms of technology (e.g. social media) and engagement seems over generalised as if social media refers to one form of technology.
- The alignment of student engagement with technology to learning outcomes is ill-defined and under-researched.
- The various forms of engagement (e.g. behavioural, emotional, social, cognitive) are not in concert with current and emergent forms of technologies.
- Current research on student engagement has not covered  typologies of the various forms of learning theories and how these can guide different forms of engagement in a technology-enhanced learning environment.
- Most studies reviewed measure student engagement through perception data; however, different data types such as trace data can provide further insights.

## Data Availability

Not applicable.

## Abbreviations

LMS: Learning management system
Moodle: Modular object-oriented dynamic learning environment
